# Pathogen reduction/inactivation of products for the treatment of bleeding disorders: what are the processes and what should we say to patients?

**DOI:** 10.1007/s00277-017-3028-4

**Published:** 2017-06-18

**Authors:** Giovanni Di Minno, David Navarro, Carlo Federico Perno, Mariana Canaro, Lutz Gürtler, James W. Ironside, Hermann Eichler, Andreas Tiede

**Affiliations:** 1 Dipartimento di Medicina Clinica e Chirurgia, Regional Reference Centre for Coagulation Disorders, Federico II University, Via S. Pansini 5, 80131 Naples, Italy; 2 Department of Microbiology, Microbiology Service, Hospital Clínico Universitario, School of Medicine, University of Valencia, Valencia, Spain; 3 Department of Experimental Medicine and Surgery, University of Rome Tor Vergata, Rome, Italy; 4 Department of Hemostasis and Thrombosis, Son Espases University Hospital, Palma de Mallorca, Spain; 5 Max von Pettenkofer Institute for Hygiene and Medical Microbiology, University of München, Munich, Germany; 6 National Creutzfeldt-Jakob Disease Research and Surveillance Unit, School of Clinical Sciences, University of Edinburgh, Western General Hospital, Edinburgh, UK; 7 Institute of Clinical Hemostaseology and Transfusion Medicine, Saarland University Hospital, Homburg, Germany; 8 Department of Hematology, Hemostasis, Oncology and Stem Cell Transplantation, Hannover Medical School, Hannover, Germany

**Keywords:** Pathogen, Inactivation, Virus, Removal, Blood, Clotting, Bleeding disorder, Infection risk, Patient information

## Abstract

Patients with blood disorders (including leukaemia, platelet function disorders and coagulation factor deficiencies) or acute bleeding receive blood-derived products, such as red blood cells, platelet concentrates and plasma-derived products. Although the risk of pathogen contamination of blood products has fallen considerably over the past three decades, contamination is still a topic of concern. In order to counsel patients and obtain informed consent before transfusion, physicians are required to keep up to date with current knowledge on residual risk of pathogen transmission and methods of pathogen removal/inactivation. Here, we describe pathogens relevant to transfusion of blood products and discuss contemporary pathogen removal/inactivation procedures, as well as the potential risks associated with these products: the risk of contamination by infectious agents varies according to blood product/region, and there is a fine line between adequate inactivation and functional impairment of the product. The cost implications of implementing pathogen inactivation technology are also considered.

## Introduction

Bleeding episodes can occur as a result of trauma, surgery, internal lesions (e.g. ulcers), infection (e.g. hemorrhagic fever), anticoagulant medication or the presence of inherited/acquired bleeding disorders such as coagulation factor deficiencies, thrombocytopenia and platelet function disorders. Such episodes are often prevented/treated using blood- and plasma-derived products. In previous decades, the life expectancy of repeatedly transfused patients was much shorter and mortality much higher due to a lack of effective treatments and the potential for transfusion-transmitted infections (TTIs) [1, 2]. Significant improvements in the manufacture and use of blood products have contributed to increased safety and patient survival [3, 4]. For example, the life expectancy of patients with haemophilia is now approaching that of the normal population.

Among the many improvements in the manufacture of blood products, the decreased risk of pathogen contamination is of particular importance [5]. As a result of this, the transmission of infectious agents via transfusion of blood and blood derivatives occurs very rarely nowadays in resource-rich countries (defined as countries with a gross national income per capita (GNI) ≥$12,616) [6, 7]. Recent international data on transfusion-transmitted viral and bacterial infections are provided in Table 1. In particular, the risk of transmitting hepatitis B virus (HBV), hepatitis C virus (HCV) or human immunodeficiency virus (HIV-1) transmission is minimal; HBV/HIV TTI was not reported at all in recent haemovigilance reports from France, Germany, Italy, Spain or the UK/USA, while rates of HCV and HEV were low in general (Table 1) [8–13], and also low in donations [8, 12, 14]. However, physicians still need to be knowledgeable about the potential risks of TTI to optimize product use, provide accurate information to patients and take appropriate action in case of suspected infection.

**Table 1:** Recent haemovigilance data on transfusion-transmitted infection

Country	Year	Infection type	Number of incidents	Implicated blood components, where known (number of cases)	Causative agents, where known (number of recipients)	Frequency over reporting period	Overall frequency of adverse transfusion-related reactions
France [8]	2015	Bacterial	2 Definite, 3 suspected	RBC (1), platelets (4)	*Staphylococcus aureus* (1—definite), *Citrobacter koseri* (1—definite); *Staphylococcus epidermidis*, non-specific *Staphylococci*	TTBI over 2015, per 10^5^:1.31 (PC), 0.04 (RBC)	241.7 per 10^5^ units transfused
		Viral	2 Definite, 1 suspected	Platelets	HEV (3)	TTVI over 2015, per 10^5^, 0.98 (PC)	
Germany [9]	2013–2014	Bacterial	11 Confirmed	RBC (5), platelets (6)	Various—from *Staphylococcus* to *Klebsiella*	Reporting frequency of TTBI for transfused units over 2013–2014, per 10^6^:7.75 (RBC), 0.97 (PC) and 1.67 (FFP) TTBI (total) over 2013–2014 per 10^6^: [5] 0.65 (RBC), [6] 6.19 (PC), [6] 0 (FFP)	No information given
		Viral	5 Confirmed	RBC (1), platelets (4)	HEV (5)	Reporting frequency of TTVI for transfused units over 2013–2014, per 10^6^: 7.75 (RBC), 0.97 (PC) and 1.67 (FFP) TTVI (total) over 2013–2014 per 10^6^: [1] 0.13 (RBC), [4] 4.12 (PC), none (FFP)	
Italy [10]	2014	Bacterial only	5	RBC (3), platelets (2)	*Serratia marcescens;* not specified		Adverse reaction requiring resuscitation procedures, 1 per 1649 transfused units. Adverse reaction inducing fatal consequences, 1 per 397,965 transfused units
Spain [11]	2015	Bacterial	3 Definite, 4 suspected	Platelets	*Serratia marcescens* (3), *Corynebacterium* spp. (2) and *Staphylococcus capitis* (2)	No information given	No information given
		Viral	1	RBC	HEV		
UK [12]	2015	Bacterial	1 Definite, 1 possible, 3 indeterminate	Platelets (2—definite/possible)	*Staphylococcus aureus* (1) and *Streptococcus agalactiae/Escherichia coli* (1)	TTI over 2015 per 10^5^: 1.6	Total cases, 436.5 per 10^5^
		Viral	2 Definite, 3 investigations pending	Platelets (2—definite), cryoprecipitate	HEV (2—definite); HCV (2—pending), HEV (1—pending)		
USA [13]	2010–2012	Bacterial and protozoal	6 Definite, 2 probable, 4 possible	Platelets (7), RBC (4), not specified (1)	*Staphylococcus* spp., *Corynebacterium* spp., *Acinetobacter/Achromobacter* spp., *Babesia microti*	TTI per 10^5^: 0.3 (RBC), 1.8 (PC), 0 (plasma), 0 (cryoprecipitate)	All events, 239.5 per 10^5^. Severe, life-threatening or fatal, 17.5 per 10^5^

*HCV* hepatitis C virus, *HEV* hepatitis E virus, *RBC* red blood cells

**Fig. 1:**
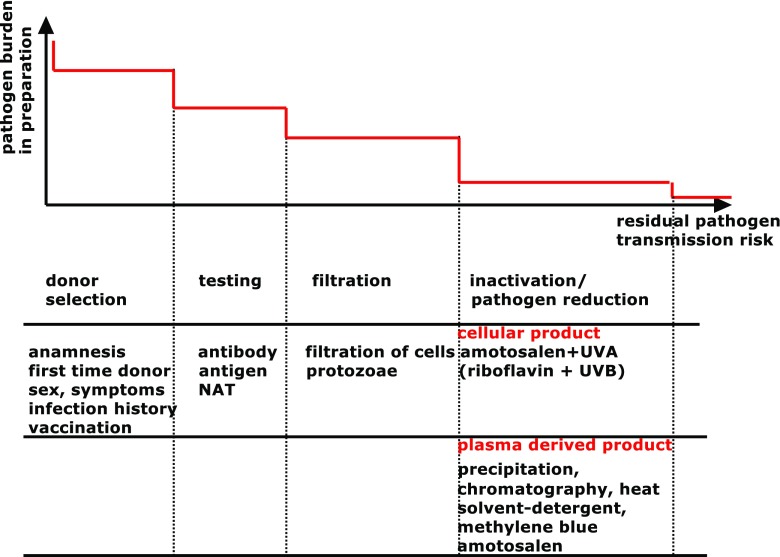
Stepwise reduction of pathogen transmission risk. Bacterial presence is routinely tested in platelet concentrates (PC) by anaerobic and aerobic cultures or by flow cytometry (discussed in the Preparation of blood-derived cellular products section). The applicability of purification and inactivation processes is limited, and is not yet possible for red blood cells (RBC). *NAT* nucleic acid testing. This figure was designed by the authors

Blood and blood-derived products that are routinely used in clinical practice include erythrocytes/red blood cells (RBC), thrombocytes/platelets (PC; for thrombocytopenia and platelet function disorders), therapeutic plasma (for multiple coagulation factor deficiencies) and plasma-derived coagulation factors (for individual/combined coagulation factor deficiencies) [15–17]. Whole blood is no longer routinely used in resource-rich countries to treat bleeding disorders, but is still occasionally used for acute bleeding episodes [16]. Separation of whole blood donations into blood-derived cellular components and plasma allows resources to be used more efficiently, since patients requiring platelets (for example) may not require RBC or clotting factor concentrates, and those components can then be redirected to the patient group where they are needed [18]. In resource-rich countries, 91% of collected blood is separated into components, compared with 72% and 31% in middle-income (GNI ≥$1036) and lower-income (GNI ≤$1035) countries, respectively [6, 7]. Separation of blood in this manner also allows for pathogen reduction/inactivation procedures to be more readily implemented. A number of steps are taken to reduce the risk of pathogen transmission: rigorous donor selection, testing for the presence of pathogens and pathogen inactivation (Fig. 1). This stepwise reduction in pathogen risk transmission improves the safety of blood-derived products, although the risk can never be altogether eradicated. There remains an overlap between adequate inactivation and degradation of beneficial components of the product [19].

The risk of pathogen contamination of blood products has fallen considerably over the past three decades, and is currently very low. For example, the residual risks for transmission of HBV, HIV-1 or HCV are approximately 1 in 500,000, 1 in 2–4 million and 1 in 8–10 million respectively in resource-rich countries [20–22]*.* However, it is important to note that the level of contamination risk varies according to country and blood product [16]; the emergence of new regional pathogens (e.g. Zika virus, which has already caused transfusion-transmitted infection in Brazil [23]) is unpredictable and could cause harm before local authorities develop means to detect and prevent blood-borne transmission. This is exacerbated by the fact that the implementation of pathogen reduction/inactivation methods varies between countries, due to differences in national blood collection methods, local resources and guidelines.

When considering the best course of treatment for patients with bleeding episodes, physicians should consider patient’s age, treatment urgency and also the potential risk of TTI. This suggests a need for physicians (and other health care professionals) to have a good understanding of the source and mode(s) of preparation of the products that they use to treat their patients, including details of blood component production (e.g. plasma-derived or recombinant factors) and pathogen reduction/inactivation techniques. This review describes the types of blood and plasma-derived products available and briefly discusses the different pathogen reduction/inactivation procedures used in their preparation. Recommendations for communicating this information to patients when explaining risks and benefits of treatments are also discussed.

## Methodology

The PubMed database was searched over the date range January 2000–February 2017, using the search strings ‘pathogen AND inactivation AND blood’. Additionally, the search terms ‘reduction’, ‘safety’, ‘haemophilia’, ‘hemophilia’, ‘clotting disorders’, ‘component inactivation’, ‘blood pathogen transmission’ and ‘blood AND product AND inactivation’ were applied. Individual searches were conducted for information on different aspects of blood collection and product treatment. Relevant references were added by the authors, who also provided additional information, experiences and opinions which were incorporated in the text.

## Separation of blood into components for treatment of bleeding disorders

Whole blood is separated as needed into cellular products, including RBC, PC and granulocyte concentrates (GC). Additional products are fresh frozen plasma (FFP) and plasma-derived products, including cryoprecipitate (rarely used in resource-rich countries at the current time) and clotting factor concentrates (e.g. fibrinogen, prothrombin complex and factors such as VIII, IX and XIII). Albumin and immunoglobulin concentrates are not routinely involved in the treatment of bleeding disorders, and do not transmit infectious agents; therefore, these are not further discussed [15–17, 24, 25].

### Preparation of blood-derived cellular products

**Fig. 2:**
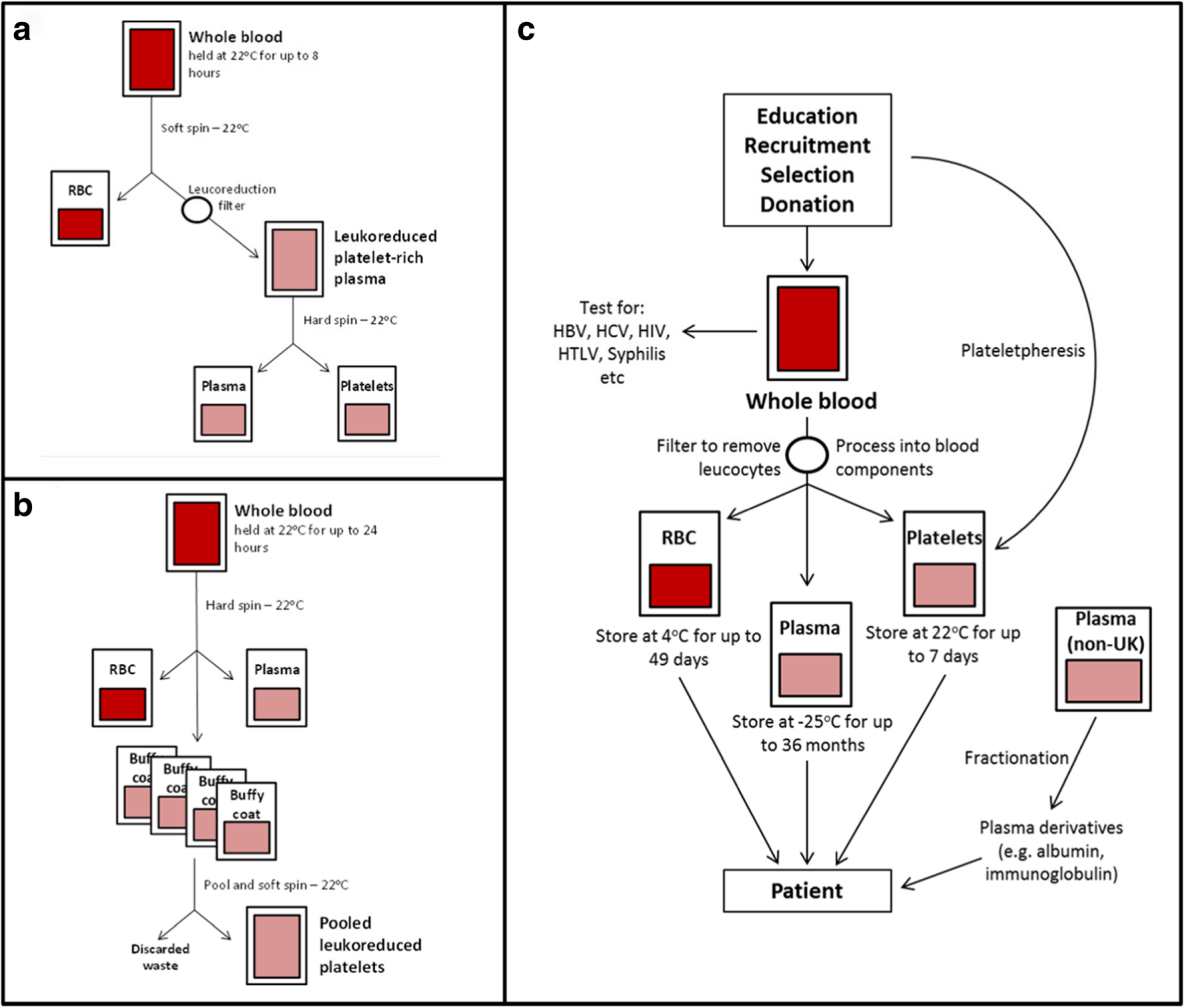
Schematic depicting methods for the separation and storage of blood-derived cellular products and plasma. **a** Platelet-rich plasma is produced by separation of RBC followed by leukoreduction. **b** Buffy coat is obtained after separation of plasma and the platelet and leukocyte enriched cell fraction from RBC either from one individual or from pooling several donations followed by leukoreduction to get PC. **c** General overview of blood processing after donor selection and testing. RBC is always provided by individual donation, while a pool of 4–6 blood donations or plasmapheresis is used for preparation of PC. Between 1000 and >10,000 plasma donations are pooled for protein preparation as FVIII and FIX [24, 25]. Non-UK plasma is used in all countries to avoid the risk of prion contamination; in the UK, non-UK plasma is used for patients born after 1 January 1996 [24]. *HBV* hepatitis B virus, *HCV* hepatitis C virus, *HIV* human immunodeficiency virus, *HTLV-1* human T-lymphotropic virus type 1. This figure was adapted from the Handbook for Transfusion Medicine, 5th Edition [24] and Vassallo and Murphy 2006 [25]

The process of separating whole blood donations into cellular components, with details of particular separation methods, is shown in Fig. 2; the different storage conditions for each blood-derived product are indicated in this figure. Whole blood donations from the UK are not processed in some countries (e.g. Australia, USA) due to the potential risk of prion contamination [26, 27].

Storage methods for whole blood (also known as ‘fresh’ blood) and PC vary. Whole blood can be stored for 24 h at a temperature of 20–24 °C [28–30], but is no longer utilized for transfusion in resource-rich countries due to the potential risk due to bacterial transmission and post-transfusion hypervolemia. The process of separation of whole blood should therefore be started within 24 h of collection (as detailed in Fig. 2). PC may be stored for 4–5 days at 20–24 °C, buffered and under permanent motion to preserve cell viability [24, 31–33]. It should be noted that storage of PC at this temperature will increase the risk of bacterial growth, as PC are at risk of bacterial contamination from donor bacteraemia or skin flora.

The initial step of whole blood processing by centrifugation involves the separation of cells based on their density, yielding RBC, white cells (buffy coat) and plasma [34]. Following centrifugation of whole blood, two methods are routinely used to continue the separation process—the buffy coat method and the platelet-rich plasma (PRP) method. The buffy coat method involves the separation of whole blood by high-speed centrifugation into RBC, white cells/platelets and plasma, while the PRP method involves the low-speed centrifugation of whole blood, leaving a larger proportion of platelets suspended in plasma. This platelet-rich plasma is then separated from the other blood components [16, 34].

Following the centrifugation of non-filtered whole blood, RBC and plasma are separated from the buffy coat, resulting in leukoreduction (i.e. total leukocyte number ≤0.1–1.2 × 10^9^ per bag). The leukocyte level of plasma and RBC can then be lowered further by an additional filtration step, after which they are referred to as leukodepleted (i.e. total leukocyte number <1.0 × 10^6^ per unit) [16]. The process of leukodepletion is mandatory in most resource-rich countries; the individual components prepared from leukodepleted whole blood do not require further leukocyte removal procedures [16].

Removal of leukocytes can reduce the risk of pathogen transmission, as leukocytes are potential carriers of pathogens such as cytomegalovirus (CMV), human T-lymphotropic virus type 1 (HTLV-1), proteinaceous infectious agents (prions) and bacterial species such as *Listeria* and *Coxiella*. The process of leukocyte removal also reduces the risk of transfusion-associated lung injury (TRALI) and alloimmunization to human leukocyte antigens (HLA) in recipients [5, 16].

#### Pathogen transmission risk of blood-derived cellular products

Common strategies for reducing the risk of pathogen transmission include (a) using a pre-donation sampling bag during initial blood withdrawal to reduce skin flora contamination [35], (b) shortening the maximum permitted storage time for PC from 5 to 4 days, (c) subjecting stored PC to screening tests for the presence of bacterial contamination after 24 h (e.g. culturing under aerobic and anaerobic conditions, or using flow cytometry [36–38]) and (d) carrying out pathogen reduction/inactivation procedures, e.g. treatment with amotosalen/UVA [4] or other agents. However, these measures are not always sufficient to prevent pathogen transmission; bacterial contamination has previously been responsible for fatal septic transfusion reactions, even when present at concentrations undetectable to screening methods (e.g. *Staphylococcus epidermidis)* [39].

It should also be noted that plasma may contain various viral species (such as hepatitis E virus (HEV), pegivirus, picornaviridae, etc. [40]) which are not routinely screened for. The relevance of these pathogens to human health is well defined in some cases, but still under investigation in others. Therefore, pathogen inactivation/reduction procedures are recommended before such products are utilized further [41, 42]. As previously noted, the processes of leukoreduction/leukodepletion reduce the risk of pathogen transmission, but do not eliminate it [16].

### Preparation of plasma-derived products

Cell-free plasma is usually frozen at −25 °C within 24 h of collection and is then referred to as fresh frozen plasma. FFP may be stored for 36 months, during which time it can be unthawed (usually at 4 °C) for use in transfusion [24, 33]. FFP can also be quarantined for several months, at which point the donor is re-screened to confirm the absence of pathogens; this procedure is preferred in some blood banks (e.g. in Germany) as it overcomes the problem of donation in the diagnostic window period when infection is not yet detectable by antibody/nucleic acid testing [43] and to avoid loss of clotting factor activity by the inactivation process. Plasma-derived coagulation factors are prepared from pooled donations by a multi-step procedure including precipitation and chromatography. The prepared factors might be kept for up to 10 days at 1–6 °C, but are usually lyophilized and can be stably maintained in this state for 6–36 months at 4 °C, or for a shorter duration if stored at 25 °C [31, 32].

#### Pathogen transmission risk of plasma-derived products

Plasma may contain a wide variety of micro-organisms, including viruses, bacteria, protozoa, microfilaria and prions; there is potential for viral and bacterial pathogens to be retained within FFP, cryoprecipitate or less frequently purified coagulation factors. The processes of filtration, freezing/thawing and component isolation (e.g. fractionation of coagulation factors) reduce the pathogen load, but do not completely remove all potential contaminants [19]. Therefore, inactivation processes are considered to be essential to enhance the safety of these products [44].

### Preparation of recombinant clotting products

Aside from plasma-derived clotting factors, recombinant concentrates have been available for factors VIIa, VIII, IX and XIII since the early 90s [45–50] and a recombinant von Willebrand factor (VWF) concentrate has recently been approved by the FDA [51]. Recombinant coagulation factors are stored in a lyophilized state and can maintain full activity for up to 30 months at 5 °C (reduced activity maintained up to 30 °C) [44]. Recombinant products are not derived from human blood, but are cloned proteins, maintained in the presence of stabilizers, such as albumin or other substances, that may be added during manufacture [52–54]. Recombinant products are classed according to the level of exposure to human plasma or albumin during their preparation. The most recently produced recombinant products (‘third-generation’ products) are produced in the complete absence of blood-derived components [45–47, 55].

#### Pathogen transmission risk of recombinant products

There are no known cases of pathogen transmission via the use of third-generation recombinant products, although external contamination of these products might still occur [56]. Recombinant products are therefore subject to at least one pathogen inactivation procedure. Processes such as chromatography, filtration/nanofiltration, solvent-detergent (SD) and dry heat treatment are regularly used in the manufacture of recombinant products and contribute to inactivation of pathogens that might be present [52, 54, 57].

*What should we say to the patient* *?* It should be noted that in resource-rich countries, the application of cellular and plasma-derived products in blood transfusion has never been safer than it is today, even if no inactivation procedures are conducted. Purified clotting factors (whether plasma-derived or recombinant) produced in these countries have very limited potential for microbial transmission. However, the risk of prion transmission via pooled plasma preparations cannot be totally discounted.

## Techniques for pathogen reduction/inactivation

As discussed, blood safety has improved with the implementation of more stringent donor selection and antibody/nucleic acid testing. The World Health Organization (WHO) recommends that all blood donations are screened for the presence of HIV, HBV, HCV, syphilis and further pathogens as required, e.g. HTLV-1 [19]. Unfortunately, the completion of questionnaires by donors cannot prevent transmission of an infectious agent if donors are unaware that they are infected [58]. Therefore, current blood collection procedures include a sampling step where donated blood is screened for pathogen contamination; donated blood which tests positive at this stage is excluded from transfusion. There is currently no routine method for inactivation of pathogenic micro-organisms in whole blood [4]; however, a recent study performed in Ghana showed efficient inactivation of *Plasmodium falciparum* in transfusion bags treated with riboflavin + UVB light, indicating that there is hope for progress in this area [59].

Measures to reduce or inactivate pathogens in blood- and plasma-derived products vary in their complexity. One simple method relies upon postponing refrigeration of donated whole blood, which allows microbes to be phagocytosed and destroyed by leukocytes within the donation bag; leukocytes are subsequently removed from the bag via filtration [16]. Storage of blood at 4 °C for longer than 24 h will inactivate some bacterial species (e.g. *Treponema pallidum*), but other species (e.g. *Listeria monocytogenes*, *Yersinia pseudotuberculosis*) are still capable of growth at this temperature and may induce septic shock when transfused with RBC [60, 61]. Therefore, the refrigeration method does not fully eradicate pathogenic contamination.

### Chemical and photodynamic treatment procedures

Chemical/dynamic treatments rely upon the interaction of a chemical agent with nucleic acids; this interaction can be triggered by UV light (e.g. amotosalen [S59] or riboflavin) or adaptation of pH and glutathione (e.g. amustaline [S303], a nucleic acid alkylator similar to amotosalen [62, 63]). This interaction causes irreversible damage to nucleic acids, thereby reducing the level of viable pathogenic contaminants [64, 65]. Photochemical (PhC) methods employ chemical agents that form a permanent linkage with RNA/DNA when exposed to light, whereas photodynamic (PhD) methods use chemical agents that simultaneously damage DNA and impede DNA repair processes [42, 63, 65, 66]. PhC and PhD technologies vary in their ability to reduce the level of pathogen contamination. They are generally effective against enveloped viruses, bacteria and protozoans, but are less reliable for the inactivation of non-enveloped virus; bacterial spores and prions are not affected by these methods. The formation of relevant neoantigens in plasma proteins has not been observed after PhC/PhD treatment [67–72].

#### Amotosalen/UVA light

This procedure (INTERCEPT™; Cerus, USA) employs the combination of a photosensitive agent (amotosalen) and UVA light and can be used for blood-derived cellular products as well as plasma and plasma-derived products. It is currently available in Europe, Australia and North America, and is the only pathogen reduction system licenced in those countries [69, 72]. After the inactivation process, the amotosalen must be completely removed, due to its genotoxicity once exposed to UVA light [73]. Studies have shown that both plasma and platelets may be safely and effectively treated with amotosalen/UVA [71, 74–77], and several countries utilize this method for pathogen inactivation of plasma [78]. The method has recently been shown to inactivate Dengue and Zika virus [77, 79]. Results from prospective randomized studies have raised questions about the ability of amotosalen + UVA treatment to reduce pathogen transmission and its impact on platelet function [80–82], as the life span and clot formation of platelets might be impaired. Their relevance after PC transfusion is still a subject of discussion [83, 84]. This is discussed further in the ‘Risks associated with pathogen reduction/inactivation treatments‘ section

#### Amustaline (S303)/glutathione

A combination of the chemical S303 and quencher glutathione can be used to treat RBC components. S303 is designed to intercalate into nucleic acids and react with DNA/RNA bases prior to breaking down to the non-reactive product S300 at neutral pH; S300 is then bound by glutathione, quenching the reaction [62]. This method is effective for platelet treatment and has been shown to successfully inactivate the Zika virus in whole blood [85] but is still under development in clinical trials [86, 87].

#### Riboflavin/UVB light

The Mirasol™ Pathogen Reduction Technology System (Terumo BCT, USA) uses riboflavin and UVB light to treat blood-derived cellular products as well as plasma and plasma-derived products. After the inactivation process, riboflavin (vitamin B2) does not need to be removed from the treated product. The procedure is safe and is not toxic or carcinogenic [65, 67, 70], but may be associated with undesirable side effects such as expression of active GPIIb/IIIa on platelets and abnormal interaction of fibrinogen [88]. This method has recently been shown to efficiently inactivate dengue virus in platelet concentrates [89], ebola virus in vitro [90] and *P. falciparum* in whole blood, as described above [59]. Riboflavin/UVB is still in the process of ongoing clinical trials [91] and has not yet been licenced.

#### Chemical/photodynamic treatment procedures specifically for plasma and plasma-derived products

##### Methylene blue + light

This was the first photochemical pathogen reduction method, described in 1991 and employs the dye methylene blue in conjunction with visible light [64, 75]. Methylene blue + light (MB-L) is still widely used for pathogen reduction of therapeutic plasma in Europe (e.g. Maco-Tronic®; Macopharma, France), but is no longer used in some countries due to concerns about the potential risk of MB accumulation and allergic reactions in multi-transfused patients [69, 75, 92].

##### UVC light

This procedure involves irradiation with UVC light alone (developed by Macopharma (France)), but the procedure is not as effective as methods employing amotosalen or riboflavin in conjunction with UV light. Moreover, this method is still in the clinical trial phase and has not been certified suitable for widespread use. The increased energy content of UVC light means that it can potentially be harmful to clotting components and may have a negative impact on coagulation [66, 69, 71].

##### Solvent-detergent treatment

The solvent-detergent (SD) process is used to treat plasma, but is not suitable for use with cellular products as the nature of the chemicals used means that the membranes of cells such as platelets or RBC are destroyed. The successful use of the SD process to reduce the level of pathogens in blood products was first described in 1986 [92–96]. During the SD process, pre-filtered plasma is treated with an organic solvent (e.g. tri-[N-butyl]-phosphate (TNBP)) and a detergent (e.g. 1% polyoxyethylene-p-t-octylphenol; Triton X-100 or Tween 80); the solvent removes lipids from viral and bacterial membranes and the detergent disrupts lipid bilayers. The combined action of the solvent and detergent therefore results in the inactivation of enveloped viruses present in the plasma [75, 94]; SD must subsequently be removed from the inactivated product, however. To further reduce the risk of blood-borne transmission of non-enveloped viruses in plasma that has undergone SD treatment, components are often purified (e.g. via chromatography), and (after lyophilization) subjected to a second inactivation step such as heat treatment [64, 97].

### Filtration/nanofiltration

These methods are used exclusively for plasma-derived purified products; after filtration with a 220-nm or 15- to 50-nm filter (filtration/nanofiltration, respectively), these products are considered to be free from bacteria and protozoans such as *Plasmodium*, *Trypanosoma* or *Leishmania.* However, contamination with small viruses and prions remains a primary safety concern [19].

### Prion reduction

There is currently no widely available reduction/inactivation method that is capable of completely removing prions from blood-derived products while maintaining functionality, but potential alternative solutions are already being implemented. Leukoreduction of red blood cells for transfusion was introduced in the UK in 1999 to reduce the risk of variant Creutzfeldt-Jakob Disease (vCJD) transmission by blood transfusion. Animal studies have shown that leukoreduction provides a high, but not complete, reduction of the risk of prion transmission by blood [98]. It is also noteworthy that the four cases of transfusion-associated vCJD transmission in the UK all occurred in recipients of non-leukoreduced RBC concentrates. More recent efforts to increase the efficiency of prion removal in blood have focused on the development of prion removal filters. However, most of these filters have been evaluated on blood spiked with hamster brain infected with a scrapie-derived prion strain (263K) [98–100]. It is highly unlikely that the physicochemical properties of these brain-derived prion spikes mimic those of prions in the blood of individuals infected with vCJD. A recent evaluation of a prion filter on blood from primates endogenously infected with vCJD found some evidence of efficacy, but vCJD transmission occurred in one primate following transfusion of ‘prion filtered’ leukoreduced RBC [99]. Further improvement and evaluation of prion filters is required, but as yet there is no initiative to introduce the use of currently available prion filters in the UK or elsewhere. The incidence of vCJD cases in the UK is declining [101], and thus the risk of prion transmission by transfusion is very low. Any residual risk might be further reduced by incorporating recent advances in vCJD prion detection in a test for blood donor screening [102, 103].

*What should we say to the patient?* There are various procedures available which are capable of inactivating protozoan, bacterial and enveloped viral species, in both PC and plasma products. Non-enveloped viruses might survive these procedures (dependent on their structure/concentration), but are not currently considered to pose any significant risk. If a high level of safety is required, inactivated products should be used for transfusion. Although a reliable means of removing prion contamination is not yet available, the overall risks of prion transmission are very low (even in the UK).

### Risks associated with pathogen reduction/inactivation treatments

The overall risk of pathogen transmission is reduced when pathogen reduction/inactivation treatments are applied to blood- and plasma-derived products. However, other risks are also associated with these treated products: key concerns include neoantigen formation, loss of product functionality/activity and the potential link between product use and adverse events in some patients [19, 64].

Neoantigens may be formed in treated blood-derived products due to changes in the three-dimensional structure of proteins, or from cellular debris. When denatured blood-derived cellular products and plasma-derived products are transfused into patients, there is a risk that the patient may develop antibodies to the neoantigens present and ultimately will be unable to maintain the course of treatment. Antibodies to therapeutic proteins are referred to as inhibitors. As a result of forming inhibitors towards a modified structure of a therapeutic protein (i.e. one which has undergone pathogen inactivation processing), there is a risk that patients may also mount an immune response against unmodified versions of the same protein, thus preventing effective replacement therapy [104], as observed, for example, in FVIII-treated patients with haemophilia A [2].

The risk of inhibitor formation with recombinant clotting factor concentrates was recently assessed in a study in 251 previously untreated patients, when patients were treated with either plasma-derived FVIII concentrate containing VWF, or recombinant FVIII to compare the risk of inhibitor formation between the two different types of FVIII products [105]. Patients receiving recombinant FVIII displayed significantly higher levels of inhibitor development compared with patients who received plasma-derived FVIII/VWF (cumulative inhibitor incidence of 44.5% vs. 26.8%, respectively; high-titre inhibitor incidence in 28.4% and 18.6%, respectively). However, the difference appeared to be maximally related to transient inhibitors: when the latter were removed from the analysis, the difference in inhibitor formation was no longer statistically significant. In addition, the results of this study differ from previously published studies which showed lower/minimal increase in inhibitor formation with recombinant FVIII [106, 107]. It is thought that differences in trial design may have played a role in this discrepancy between current and previous results.

Another limitation of pathogen inactivation processes is the reduction in the activity of products. For example, SD treatment can cause a reduction in FVIII activity in plasma-derived products; FVIII treated with SD (Octaplas®, Octapharma) has a FVIII activity level ~10–20% lower than that observed in FFP. Process validation studies conducted in three European blood centres showed that PhC-treated plasma displayed a ~26% reduction in FVIII activity compared with that of FFP [94]. The activity level of other coagulation factors was also reduced, but to a lesser extent with clotting factor activity generally reduced by less than 20% [108]. Compared with FFP, SD-treated plasma displayed reduced activity levels for VWF and Protein S (67–76% and 35% reduction, respectively), while MB-L-treated plasma displayed lower fibrinogen activity levels (approximately 20% reduction) [94, 104, 109].

There have been various reports linking pathogen inactivated products with adverse events. As mentioned, use of MB-L-treated plasma has been associated with post-transfusion anaphylactic reaction, and a study of plasma use in one hospital over several years showed that patients who received MB-L-treated plasma required 56% more plasma in total than patients who received non-inactivated plasma; the increased need for plasma in this instance was described as an adverse event, but could also potentially indicate reduced therapeutic efficacy of the MB-L-treated plasma [104]. MB-L-treated plasma, but not SD-treated plasma, may also contain residual RBC or cell fragments, which increase the risk of red cell alloimmunization; it is still unclear whether the presence of these residual elements is influenced by the nature of plasma processing prior to inactivation [104, 110–112]. Concerns have previously been raised over reports of a potential link between SD-treated plasma and increased risk of venous thromboembolism, potentially caused by low levels of Protein S, leading to an increased risk of clot formation [94, 113]. However, a recent study, and the resumption of SD-treated plasma use in the USA, indicates that the association of a potential link is still uncertain [114]. There has been no reported association between SD treatment and TRALI, which can be a major cause of transfusion-related mortality using plasma components containing leukocyte-directed alloantibodies (5–25% of cases are fatal) [20]. This is thought to be due to low levels of human leukocyte antigen antibodies, the key mediators of TRALI, in SD-treated plasma caused by the pooling and dilution process [104].

*What we should say to the patient:* Inactivation of infectious agents improves the safety profile of blood-derived products. For purified plasma proteins, two inactivation procedures (based on different inactivation principles) are usually combined. Inactivation procedures that alter the structure of components of infectious agents might also affect the natural shape of proteins and cause new epitope (neoantigen) formation on some proteins, leading to a subsequent immune reaction that may impair the activity of that protein (e.g. affecting coagulation, in the case of an enzyme involved in the coagulation cascade). All pathogen inactivation procedures have some unwanted effects on the product; on the other hand, lack of treatment may have fatal consequences due to the survival of infectious agents. These situations must be evaluated individually, while still considering the possibility of (rare) side effects.

## Cost considerations

Ideally, pathogen reduction/inactivation should be widely effective, inexpensive and not cause any reduction in the functionality or activity of the product(s) [69]. Practically, the aim is to reduce the pathogen transmission risk of the final product as much as possible while avoiding a significant loss of clotting factor activity which could result in an increase in cost. The potential cost of treating conditions caused by, or linked to, pathogen reduction/inactivation procedures should also be considered [104]. It is difficult to predict the costs associated with any newly emerging diseases, as there are many factors to take into account (including the cost of morbidity/mortality, loss of earnings, reduction of productivity, etc). For example, retrospective analyses of the economic impact of infectious diseases such as dengue fever have not yet provided a clear picture of their overall cost [115, 116]. Expenditure on medical research should also be considered. The detection of emerging viruses and the development of an effective response to reduce the risk of transmission (e.g. West Nile Virus in 1999, Chikungunya in 2008, Zika virus in 2016) has resulted in additional expenditure on research and diagnostics, and has also historically been responsible for the loss of blood donations from affected regions [117–119].

Logically, routine implementation of pathogen reduction/inactivation technology would lower the risk of infection caused by novel blood-transmissible pathogens. Furthermore, if highly effective pathogen reduction/inactivation procedures were in place, there would be reduced need for additional expenditure on research and the loss of donors and their donated blood could be kept to a minimum [64, 92]. It must however be noted that although routine implementation of pathogen reduction/inactivation technology would (theoretically) lower the risk of transmission of novel blood-borne pathogens, the cost-effectiveness of implementation and potential risks to the quality of life of the recipient should also be taken into consideration. The main benefit of pathogen inactivation procedures is the prevention of bacterial growth/reduction of sepsis risk, in addition to CMV inactivation and prevention of alloimmunization by HLA proteins or platelet glycoproteins. The main impediment is, as discussed, the loss of platelet activity and clotting capacity (up to 60%); however, this has not been reported to have any impact either in acute transfusion reactions [74] or post-transfusion bleeding [120].

Transfusion reactions occur less frequently in amotosalen/UVA-inactivated PC [81], and the rate drops further when PC are stored in a platelet additive solution III; rates of bleeding events in recipients of these buffered platelets were lower versus recipients who received amotosalen/UVA-treated platelets in the same buffer, while the occurrence of infections was not different [80]. These findings indicate that, in selected donor populations and unstandardized PC preparation conditions, there is only a tiny difference in likelihood of infectious agent transmission between inactivated and non-inactivated preparations. The cost balance therefore has to be individualized for each recipient and his/her health status [80, 121]. Finally, the benefits of lowering infection/alloimmunization risk and likelihood of higher individual bleeding risk is difficult to assess in monetary terms. The value of an individual life is not quantifiable in any currency, and cannot be negotiated.

### Cost-effectiveness of pathogen inactivation/reduction technologies

There have been several studies investigating the cost-effectiveness of pathogen reduction/inactivation. In one report (based on the UK system), the costs per life year saved for patients aged ≤60 years using three pathogen inactivation systems (involving amotosalen/UVA, riboflavin/UVB or UVC treatment) were estimated to range from £3.4–9.1 million [122]. A retrospective Spanish study showed that universal implementation of pathogen reduction (amotosalen/UVA system) for platelet preparation from 2008 onwards extended platelet storage time and reduced the cost associated with out-of-date units, resulting in a saving of 13.8% of the budget compared with the initial combined cost of platelet production/storage/wastage for one institution [123]. In a Belgian study, implementation of an amotosalen-based pathogen reduction system for platelets was found to be cost-effective compared with the initial national system (measured in terms of lifetime costs and quality adjusted life years (QALYs)). A range of incremental cost-effectiveness ratios were observed (in absence of emerging pathogens) from 3,459,201 €/QALY to 195,364 €/QALY), and the mean threshold of emerging infection risk for the pathogen reduction system ranged from 1/1079 to 1/2858 transfusion [124]. In addition, a model that was developed from a Dutch study found that implementation of pathogen inactivation as a routine procedure would be a cost-effective measure (measuring cost-effectiveness in net costs per life year gained (LYG)); net costs per LYG with pathogen inactivation were estimated at €554,000 in the baseline-weighted average over three patient groups (90% simulation interval €354,000–1092,500). A sensitivity analysis indicated that cost-effectiveness in this model was insensitive to viral risks and indirect costing, but highly sensitive to the assumption of a requirement for excess transfusions required and also discounting of life years gained. The study authors concluded that, in view of the relatively high (and internationally accepted) net costs per LYG for blood transfusion safety interventions, the data generated by this model indicate that pathogen inactivation may be cost-effective. However, the study made the assumption that the pathogen inactivation method would be 100% effective, rendering this result uncertain [125]. In Germany (for example), approximately 20 million € (50 € (assumed price) for amotosalen/UVA treatment for 400,000 PC) would have to be paid in order to prevent 6 instances of TTBI [9]. In the USA, the cost-effectiveness of pathogen inactivation was assessed at five institutions, comparing pathogen inactivation costs with the costs of tests/procedures currently performed; total potential cost savings with the implementation of pathogen inactivation were estimated at $141.65 per apheresis platelet unit [126]. In the UK, approximately £1.0–3.2 million would have to be spent in order to save 0.9–1.3 LYG per unit when using amotosalen/UVA inactivation to reduce bacterial contamination of platelets [122].

Another potential cost-saving approach would be to prepare blood components from inactivated whole blood, treated by an agent such as riboflavin/UVB; studies have shown that whole blood treated with this method is safe for transfusion use, as are its components [59, 65, 127]. This approach may have merit in countries which rely on whole blood as their main transfusion source. However, it should be noted that the process of pathogen inactivation of whole blood by riboflavin/UVB has not yet been optimized, and that some non-enveloped viruses (e.g. hepatitis A virus) may be poorly inactivated. Therefore, the overall cost-effectiveness of this approach (when the potential cost of treating patients for TTI is taken into account) has yet to be determined.

## Governing bodies for blood transfusion practices

Organizations such as the European Directorate for the Quality of Medicines and Healthcare (EDQM) support the right of access to good quality medicines/healthcare. This includes the safe/ethical ‘collection, preparation, storage, distribution and appropriate use of blood components (for) blood transfusion’ according to their recommended quality standards [29]. Within the EDQM, the relevant committees are the European Committee on Blood Transfusion (CD-P-TS) and the Committee on Quality Assurance in Blood Transfusion Services (GTS). The recommendations of these committees are in keeping with those published by the FDA [30, 32, 128]. The blood transfusion programme recommended by these committees is focused on three main principles: the importance of voluntary (non-remunerated) blood donation, the goal of self-sufficiency in meeting the demand for blood products and the need to protect both blood donors and recipients from any potential harm. However, there is currently no consensus position as to preferred pathogen reduction/inactivation technologies [31]. The EDQM has made a series of recommendations over the years designed to encourage further investigation into the safety, clinical usefulness and cost-effectiveness of pathogen reduction/inactivation technology [30, 33]. Similar recommendations were published by the FDA and AABB in 2015 [35, 129].

A report on the collection, testing and use of blood and blood/plasma components in Europe was issued by the EDQM in 2010; this report provided information on pathogen reduction/inactivation technology utilization across 33 member states in Europe (72% of all member states) [32]. Leukodepletion was carried out for all RBC-containing products, plasma for transfusion and platelet concentrates in 38% (12/32 states), 38% (9/24 states) and 56% (18/32 states) of respondents, respectively. Irradiation of blood components was also conducted in some cases (primarily to eliminate contamination with any residual living leukocytes) but irradiated products were restricted to at-risk patients only, e.g. immunocompromised individuals, who have a greater risk of developing graft versus host disease. National use of a quarantine stage and SD and MB-L treatment is also described in the report. In total, 24% (7/29 states) employed quarantine only, 19% (5/27 states) utilized pathogen reduction/inactivation technology almost exclusively and 6% (2/31 states) used a combination of the two methods [32].

## Communicating residual pathogen risk to patients

Up to this point, we have described current strategies for minimizing the risk to patients in terms of pathogen transmission, while striking an appropriate balance of maintaining efficacy and keeping processing costs within reasonable limits. An important consideration is how this information should be presented to patients when describing the risks and benefits when obtaining informed consent [130, 131] This information has to be relayed in words/terms that the patients can readily understand, so that they may become aware of all potential consequences of transfusion, preferably using an established communication strategy [130–132]. It may be beneficial to define the possible risk of transfusion in relation to risk in general (such as the risk of lightning strike [1 in10^7^] or death by road accident [1 in 8000] [132]) in such a way as to avoid any misunderstanding [130].

In general, the consent procedure for every kind of blood or plasma transfusion should include:

1. A description of the risks as well as benefits of treatment, and listing of alternative approaches (including non-treatment)

2. The opportunity to ask questions at any time

3. The right to accept or refuse the proposed treatment [29, 133, 134]

Patients should understand that despite improvements in donor screening, testing methods and pathogen removal techniques, the risk of pathogen transmission cannot be completely eliminated (as explained above). The extent to which patients should be further informed about specific risks depends on a variety of factors, including the urgency of treatment, availability of alternative treatments, the pathogenic risk associated with the particular product and, finally, patient capacity (including age, physical/mental condition, education and level of understanding, language barriers and religious beliefs). Ethical considerations of the risk of transfusion treatment must be respected as well [91, 130]. In emergency situations, administration of blood- and plasma-derived products may be justified without the patient’s consent if it is judged to be in their best interests according to available information and standards. However, patients should be informed about the potential risks of such emergency treatments as soon as possible.

For long-term treatments, such as prophylactic factor replacement or repeated transfusions, the provision of accurate information relating to pathogen transmission is of utmost importance. Information should be regularly made available, at the first treatment and all other treatment sessions. Care should also be taken to make patients aware of information updates and novel products with comparable or better pathogenic risk profiles. In their Guidelines for the Management of Hemophilia, the World Federation of Hemophilia (WFH) states that the core team providing care for a haemophilia patient must include at least one member whose role is to provide educational assistance for the patient and their family [133, 134]. In many cases, this role will fall to the physician; therefore, it follows that physicians would benefit from having access to guidance on how best to convey treatment-related information to patients [130], so that both the physician and patient (or the patient’s family) may discuss and agree upon the most appropriate course of treatment. The guidelines for providing information to patients with haemophilia vary according to region; details from selected countries are presented in Table 2. It is the recommendation of the authors that all physicians should ensure that they are fully informed and aware of local guidance before counselling patients. The current local guidelines for providing patient information are generally clear, but we feel that they would benefit from a harmonization of approach: possibly in the form of international guidelines.

**Table 2:** International/national guidelines for physicians when informing haemophilia patients of pathogen transmission risk; highlighting the variability of how this important information is handled

Geographical region/country	Guidelines	Source
World	World Federation of Hemophilia (WFH) guidelines for the management of haemophilia: On the choice of products for haemophilia and related disorders: ‘The WFH strongly recommends the use of viral-inactivated plasma-derived or recombinant concentrates in preference to cryoprecipitate [*Author’s note: cryoprecipitate harbours a high risk of viral contamination*] or fresh frozen plasma for the treatment of haemophilia and other inherited bleeding disorders.....[*Author’s note: harbours a high risk of hypervoluminemia]* When selecting plasma-derived concentrates, consideration needs to be given to both the plasma quality and the manufacturing process. Two issues deserve special consideration: Purity of product Viral inactivation/elimination’	Section 4.1, Guidelines for the management of hemophilia [134]
	On pathogen safety: ‘The new challenge remains emerging and re-emerging infections, many of which are not amenable to current risk reduction measures. These include the non-lipid enveloped viruses and prions, for which diagnosis and elimination methods are still a challenge.’	Section 6.3, Guidelines for the management of hemophilia [134]
Germany	Clinical guideline for the use of blood and blood-derived products: ‘The responsible physician must obtain the patient’s informed consent before starting a transfusion.’ Contents of patient information are not further specified.	Cross-sectional guidelines for therapy with blood components and plasma derivatives [135]
	Guideline on the preparation and use of blood and blood-derived products: (Translation) ‘Blood components and plasma derivatives are prescription items and shall be subject to medical prescription. The indication must be considered thoroughly and with careful consideration of the individual patient. […] Informed consent should be obtained at the earliest possible time in order to allow the patient sufficient time before consent.’	Section 4.3, Richtlinien zur Gewinnung von Blut und Blutbestandteilen und zur Anwendung von Blutprodukten (Hämotherapie) [43]
	(Translation) ‘If informed consent cannot be obtained, e.g. in emergency situations, patients shall be informed retrospectively about the administration of blood products and their risks, in particular infection and immunization risks.’	Section 4.3.10, Richtlinien zur Gewinnung von Blut und Blutbestandteilen und zur Anwendung von Blutprodukten (Hämotherapie) [43]
Italy	Recommendations for the transfusion of plasma and platelets ‘In all cases, it is recommended that the patient and/or his/her parents/tutors receive detailed and clear information concerning the available therapeutic approaches and replacement products, in order to achieve shared decisions and the written informed consent for treatment and the choice of the product.’	Italian Society of Transfusion Medicine and Immunohaematology (SIMTI) Working Party [136]
	Patient consent: The patient consent form includes a detailed description of the different levels of pathogenic risk arising from different products for treating bleeding disorders, and requires the patients to confirm that they have understood and agreed to these risks.	Informed consent form for haemophilia treaters of Italy (Consenso Informato e informativa) [137]
Spain	Transfusion recipients are required to provide informed consent and information on pathogen risk is given within the informed consent form. However, no specific recommendations are provided pertaining to how physicians should advise patients on pathogen risk.	Estándares en Transfusión Sanguínea – Fundacion CAT (4th edition) [138] Informed consent form for transfusion (Son Espases University Hospital, Spain) [139]
UK	Patient information and consent for transfusion ‘Where possible, patients (and for children, those with parental responsibility) should have the risks, benefits and alternatives to transfusion explained to them in a timely and understandable manner. Standardized patient information, such as national patient information leaflets, should be used wherever possible’	Table 4.1, Handbook of transfusion medicine (5th Ed) [24]
	The Advisory Committee on the Safety of Blood, Tissues and Organs (SaBTO) recommends that ‘valid consent’ for blood transfusion should be obtained and documented in the clinical record. The following recommendations apply: ‘Use of standardized sources of information for all patients in the UK – appropriate information leaflets are available from the UK Transfusion Services and should be used in all hospitals.’	Section 4.4, Handbook of transfusion medicine (5th Ed) [24]
	‘Use of a standardized information resource for clinicians, indicating the key areas to be discussed when obtaining consent – an example is available from http://www.transfusionguidelines.org.uk/index.asp?Publication=BBT&Section=22&pageid=7691.’ ‘Patients needing long-term transfusion support should have a modified form of consent (e.g. annual review and re-consent) and this should be specified in local policies.’	http://www.transfusionguidelines.org.uk/index.asp?Publication=BBT&Section=22&pageid=7691
US	Guidance on informed consent for blood transfusion: ‘…..at a minimum, elements of the consent shall include the following: (1) a description of the risks, benefits, and treatment alternatives (including non-treatment), (2) the opportunity to ask questions, and (3) the right to accept or refuse transfusion.’	Friedman et al. [140]
	Guidance on blood donation: ‘Elements of the donation procedure shall be explained to the prospective donor in understandable terms. The explanation shall include information about risks of the procedure, tests performed to reduce the risks of transmission of infectious diseases to the allogeneic recipient and requirements to report donor information, including test results, to state or local health departments. The donor shall have an opportunity to ask questions and have them answered and to give or refuse consent for donation. In the case of a minor or a legally incompetent adult, consent shall be addressed in accordance with applicable law.’	AABB standards for blood banks and transfusion services, 26th edition [141]
	Information regarding attitudes, practices and training on informed consent for transfusions and procedures: ‘60% of respondents (medical students and physicians in the US) felt that their informed consent training was adequate. Multiple areas of difficulty in obtaining proper informed consent were identified that should be addressed with focused training or written guidelines.’	Vossoughi SR et al. [142]

## Conclusion

Since the residual risk of transmission of a blood-borne infectious agent by blood, blood-derived cellular products or plasma-derived products is never zero, it is prudent to reduce the risk of transmission as far as possible. Only accepting blood from pre-screened donors is the first step towards reducing risk, but further processing measures are still required, as unknown pathogens may still potentially be present or newly emerge. Selection of appropriate therapeutic products can also help to reduce the residual risk, i.e. choosing a blood component that is less likely to harbour a pathogen, or selecting a product which has not been exposed to blood or plasma at all. There are a variety of effective pathogen inactivation/reduction procedures available. Implementation of such procedures therefore has to be balanced by considerations of efficacy, health benefit and potential side effects and, finally, financial perspectives.
